# Tumor-infiltrating lymphocyte subsets and tertiary lymphoid structures in pulmonary metastases from colorectal cancer

**DOI:** 10.1007/s10585-016-9813-y

**Published:** 2016-07-23

**Authors:** Thomas Schweiger, Anna Sophie Berghoff, Christoph Glogner, Olaf Glueck, Orsolya Rajky, Denise Traxler, Peter Birner, Matthias Preusser, Walter Klepetko, Konrad Hoetzenecker

**Affiliations:** 1 Division of Thoracic Surgery, Department of Thoracic Surgery, Medical University of Vienna, Waehringer Guertel 18-20, 1090 Vienna, Austria; 2 Christian Doppler Laboratory for Cardiac and Thoracic Diagnosis and Regeneration, Medical University of Vienna, Vienna, Austria; 3 Department of Medicine I, Medical University of Vienna, Vienna, Austria; 4 Comprehensive Cancer Center, Medical University of Vienna, Vienna, Austria; 5 Department of Pathology, Medical University of Vienna, Vienna, Austria

**Keywords:** Pulmonary metastasis, Colorectal cancer, Tumor-infiltrating lymphocytes, TILs, Tertiary lymphoid structures

## Abstract

The presence of tumor-infiltrating lymphocytes (TILs) and tertiary lymphoid structures (TLSs) reflects an active inflammatory tumor microenvironment. High density of TILs as well as presence of TLS is associated with improved survival in various solid cancer types. We aimed to describe the density and distribution of TILs and TLS in pulmonary metastases (PMs) from primary colorectal cancer (CRC) and its correlation with clinicopathological variables. Fifty-seven CRC pulmonary metastasectomy specimen (PM) and 31 matched primary CRC specimen were included. Cluster of differentiation (CD)3+, CD8+, CD45RO+ and FoxP3+ TILs were evaluated by immunohistochemistry and density was scored semiquantitatively. TLS were evaluated based on morphological criteria. Survival time was defined from pulmonary metastasectomy to death or last follow up. A marked infiltration with CD3+, CD8+, CD45RO+ and FoxP3+ TILs was evident in CRC PM and matched primary CRC. Further assessment of the immune infiltrate in PM showed that a high density of FOXP3+ TILs at the invasive margin [HR 2.40 (1.11–6.96); *P* = 0.031] and low density of CD8+ cells in TLS [HR 0.30 (0.14–0.79); *P* = 0.016] were associated with a worse prognosis in univariate analysis. Moreover, a low CD8/FoxP3-ratio of TILs at the invasive margin (*P* = 0.042) and in TLS (*P* = 0.027) conferred an impaired prognosis after pulmonary metastasectomy. Our findings suggest that CRC PM harbor an immune active microenvironment. The balance of CD8+ and FoxP3+ T-cells at the tumor border and in TLS provides prognostic information in patients with CRC PM.

**Electronic supplementary material:**

The online version of this article (doi:10.1007/s10585-016-9813-y) contains supplementary material, which is available to authorized users.

## Background

Despite advances in the early detection and treatment of colorectal cancer (CRC), the prognosis of patients is impaired as soon as distant metastases occur. Synchronous pulmonary spreading is evident in about one out of ten patients with newly diagnosed CRC. Subsequently, an average 5-year cumulative risk of 5.8 % for the development of metachronous pulmonary metastases (PMs) is additionally contributing to the disease burden of patients with CRC [1]. However, within the group of patients with CRC lung metastases long-term survival can be achieved by (repeated) pulmonary metastasectomy complemented by chemotherapeutic regimens. In contrast, some patients will present with diffuse recurrence of disease within months after pulmonary metastasectomy. The underlying tumor biology is considered to be the main cause for heterogeneity in the outcome of patients with CRC lung metastases. Several prognostic biomarkers have been proposed to define aggressive tumors associated with fatal outcome [2–4]. During the last years, the tumor microenvironment gained increasing attention in the scientific community, especially in groups focusing on metastatic CRC [5–7].

Immune escape is considered an emerging hallmark of cancer [8]. Various subsets of lymphocytes can be found in the tumor microenvironment, so called tumor-infiltrating lymphocytes (TILs). They can launch pro-inflammatory anti-tumor responses or mediate local immunosuppression. The amount of TILs has a prognostic value in various primary solid cancer types, including lung, renal, breast and CRC [9–14]. Commonly detected lymphocyte subsets with favorable prognostic impact are mature T-cells [cluster of differentiation (CD)3+] and cytotoxic T-cells (CD8+), memory-T-cells (CD45RO+), while immune suppressive regulatory T-cells (FoxP3+) are associated with impaired prognosis. Moreover, tertiary lymphoid structures (TLSs), which are ectopic lymphoid aggregates present in chronically inflamed tissue, can be found in the tumor stroma. TLS are believed to promote and maintain inflammation and anti-tumor response similar to secondary lymphoid organs. The presence of TLS in the tumor microenvironment is associated with favorable prognosis especially in CRC [15, 16].

So far little is known about the local immune response in CRC metastases, as previous studies focused on primary tumor specimen. Metastasis initiating cells have already successfully conquered immune escape during intravasation, survival in the blood stream and extravasation indicating that the composition of the immune microenvironment might differ from the primary tumor [17, 18]. Therefore, we aimed to investigate the local inflammatory microenvironment in CRC PM specimen and matched primary CRC specimen.

## Materials and methods

### Study population

From April 2009 to June 2014 57 patients with primary CRC receiving complete/curative pulmonary metastasectomy at the Medical University of Vienna were retrospectively recruited from a prospective institutional database. The study was approved by the Institutional Ethics Committee (# 1035/2014) and conducted according the Declaration of Helsinki. Resected pulmonary nodules were verified as metastases from primary CRC by a board certified pathologist. Additionally, samples of the corresponding primary tumor were available in 31/57 (54.4 %) patients. The patients underwent post-surgical tumor surveillance after pulmonary metastasectomy including periodical computed tomography (CT) scans.

### Immunohistochemistry

Immunohistochemical (IHC) staining was performed on 4 μm thick sections of formalin-fixed, paraffin-embedded tissue samples using an automated staining platform (Ventana Benchmark Ultra immunostainer, Ventana Medical Systems, Inc., Tucson, USA).

Immunostaining was performed with anti-CD3 (clone SP7, #RM9107-S1, Thermo Fisher Scientific, Cheshire, UK), anti-CD8 (clone C8/144B, #M7103, Dako, Glostrup, Denmark), anti-CD45RO (clone UCHL1, #M074201, Dako, Glostrup, Denmark) and anti-FOXP3 (clone 206D, #320116, BioLegend, San Diego, CA, USA) antibodies using an autostainer (Benchmark Ultra, Ventana Medical Systems, Tucson, USA) according to the manufacturer’s instructions. In negative controls the primary antibody was omitted. A mediastinal lymph node served as positive control. The presence of lymphatic vessel invasion in PM was known from a previous study [19].

### Quantification and scoring of TILs and TLS

The density of CD3+, CD8+, CD45RO+ and FoxP3+ TILs was evaluated in a semiquantitative manner on full size sections as described previously [20, 21]. In summary, scores reaching from 0 (absent infiltrate) to 4+ (very dense infiltrate) were assigned to the tissue samples. First, an overall impression was rated at low magnification (×100). Additionally, the spatial distribution of the immune infiltrate was assessed in the tumor center and at the invasive margin separately at higher magnification (×200–400). For further analyses, the TILs densities were dichotomized. The used cut-offs are provided in Supplementary Table 1. Moreover, the sections were screened for the presence of TLSs based on morphologic features (TLS; present vs. not present). Follicular aggregates of lymphatic cells were defined as TLS, whereas perivascular lymphatic aggregates were excluded. The presence of CD3+, CD8+, CD45RO+ and FoxP3+ cells in the TLS was assessed and, according to TILs quantification, scores from 0 to 4 were assigned to the samples. In general, negative and sparse infiltration (0 and 1+) were grouped in contrast to moderate, dense and very dense infiltration (2+, 3+ and 4+), which led to two group sizes as equal as possible. As CD3 were omnipresent, a higher cut-off had to be applied. Also for CD8 TILs, a higher cut off was chosen as described previously by others [22, 23]. The dichotomized variables were used for further calculations. CD8/FoxP3 ratios were calculated from dichotomized values. Samples with high CD8/low FoxP3 were described as CD8/FoxP3-ratio high, samples with high CD8/high FoxP3 or low CD8/low FoxP3 as equal and low CD8/high FoxP3 were described as low. Two observers (TS, ASB) blinded to the clinical data rated each section independently using a multi-head microscope. If the rating differed, the slide was re-discussed and a consensus was found.

### Statistical analysis

The disease-free interval (DFI) was defined as the time between surgery for the primary tumor to pulmonary metastasectomy in months. Time to recurrence represented the time between pulmonary metastasectomy and evidence of recurrence at any organ site. Overall survival (OS) was defined as the period of time between pulmonary metastasectomy and death of any cause. If patients had a history of pulmonary metastasectomy before the inclusion period, the previously resected metastases were assessed and the outcome was calculated from the first pulmonary metastasectomy. Nominal variables were compared using χ^2^ test or Fisher’s exact test (if expected frequency <5). Survival curves were estimated using Kaplan–Meier plots and the difference of the groups were compared using the log-rank test. Statistically significant variables (*P*-values ≤0.05) in the univariate analysis were added to a multivariate Cox regression model. Statistics were performed using SPSS 23 (SPSS, Inc., Chicago, USA) and GraphPad Prism 6 (GraphPad Software, Inc., California, USA) software. All performed tests were two-sided. *P*-values ≤0.05 were considered statistically significant. Due to the hypothesis generating approach of the study no correction for multiple testing was used [24].

## Results

Fifty-seven patients with histologically verified PMs from primary CRC were included in this study. Macroscopically and microscopically complete resection was achieved in all patients. 33 (57.9 %) patients were male and 24 (42.1 %) female. Median age at the time of pulmonary metastasectomy was 64 years (range 33–83). The primary tumor site was colon in 32 (56 %) patients and rectum in 25 (44 %) patients. A detailed description of the patients’ characteristics is provided in Table 1.

**Table 1:** Demographic details of the study cohort (n = 57)

Characteristics	Total study cohort (n = 57)	
	n	%
Median age at surgery (range)	64 (33–83)	
Median follow-up after metastasectomy in months (range)	30 (4–137)	
Sex		
Male	33	57.9
Female	24	42.1
Localization of primary tumor		
Colon	32	56.1
Rectum	25	43.9
UICC stage of primary tumor		
I	4	7.4
II	14	25.9
III	27	50.0
IV	9	16.7
Unknown	3	–
Previous liver metastasis		
Yes	17	29.8
No	40	70.2
DFI		
<36 months	38	66.7
36–60 months	9	15.8
60 months	10	17.5
No. of pulmonary metastases		
Singular	37	64.9
Multiple	20	35.1
Chemotherapy before metastasectomy		
Yes	45	78.9
No	12	21.1
Chemotherapy after metastasectomy		
Yes	43	75.4
No	14	24.6

*DFI* disease-free survival to first pulmonary metastasis

### Density and distribution of TILs and TLS in pulmonary metastases

**Fig. 1:**
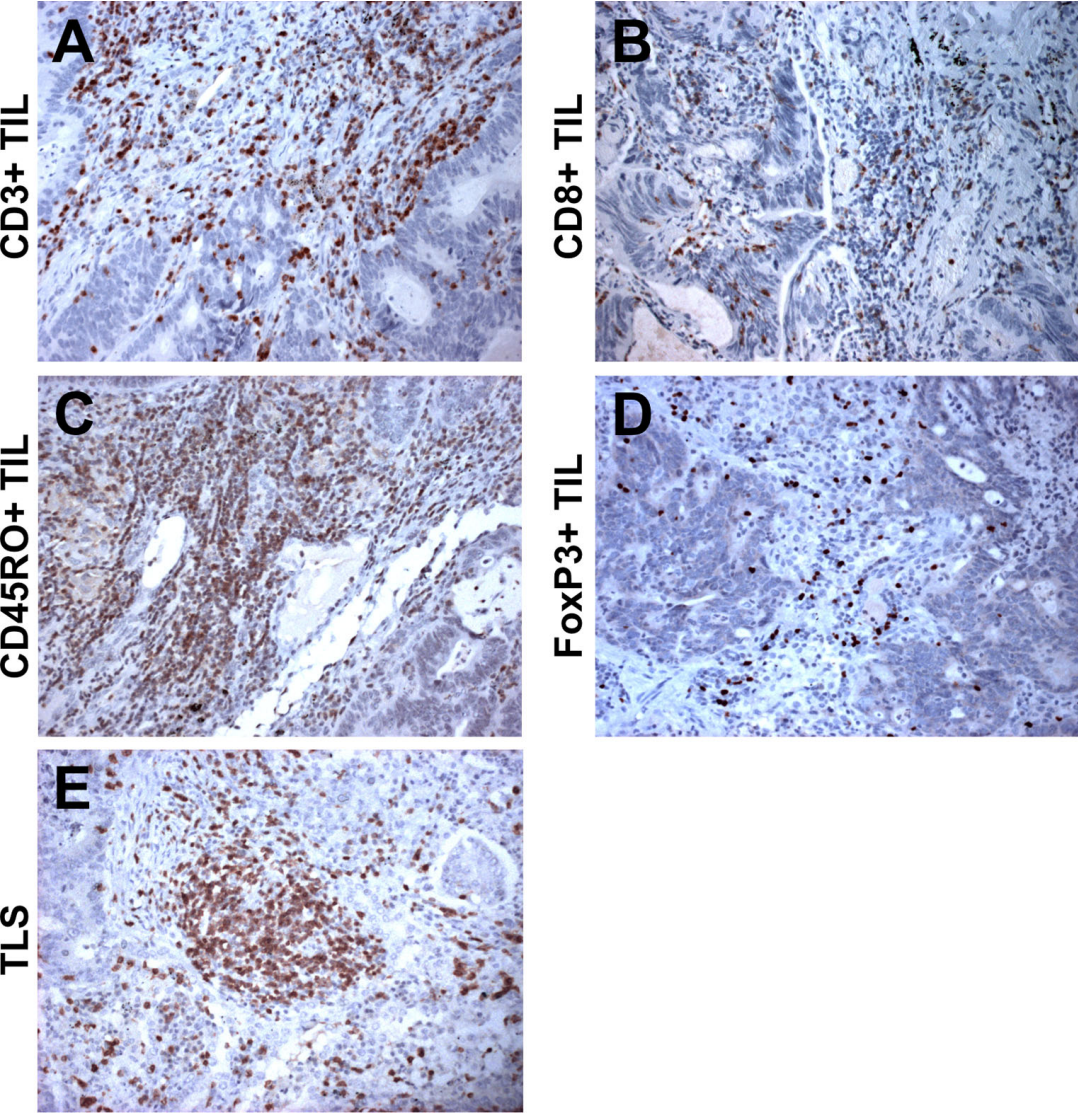
Representative *images* of high densities of **a** CD3+, **b** CD8+, **c** CD45RO+ and **d** FoxP3+ TILs (DAB; *brown*) in pulmonary metastases. **e** CD3+ tertiary lymphoid structure at the invasive margin between tumor cells and lung parenchyma (magnification ×200/400). (Color figure online)

Sufficient IHC quality was achieved in 55/57 (96.5 %), 57/57 (100 %), 54/57 (94.7 %) and 53/57 (93.0 %) of PM specimen for CD3+, CD8+, CD45RO+ and FoxP3+ TIL evaluation, respectively. CD3+, CD8+, CD45RO+ and FoxP3+ TILs at variable density were evident in 55/55 (100 %), 55/57 (96.5 %), 50/54 (92.3 %) and 45/53 (84.9 %) of PM. A detailed description of the density and spatial distribution of TILs is shown in Table 2. Representative images of TILs are provided in Fig. 1. TLS were present in 45/57 (78.9 %) PM specimen. If TLS could be found, the density of CD3+, CD8+, CD45RO+ and FoxP3+ T-cells in TLS was assessed (Supplementary Table 2) and correlated with clinicopathological characteristics (Supplementary Table 3).

**Table 2:** Semi-quantitative description of tumor-infiltrating lymphocytes in CRC pulmonary metastases

TILs density	CD3+ TILs		CD8+ TILs		CD45RO+ TILs		FoxP3+ TILs	
	n	%	n	%	n	%	n	%
Tumor center								
0	0	0.0	2	3.5	4	7.0	8	14.0
1+	7	12.3	28	49.1	18	31.6	19	33.3
2+	21	36.8	15	26.3	28	49.1	24	42.1
3+	20	35.1	12	21.1	4	7.0	2	3.5
4+	7	12.3	0	0.0	o	0.0	0	0.0
Total	55	96.5	57	100.0	57	100.0	57	100.0
Invasive margin								
0	0	0.0	13	22.8	2	3.5	10	17.5
1+	3	5.3	21	36.8	13	22.8	26	45.6
2+	20	35.1	17	29.8	32	56.1	17	29.8
3+	22	38.6	6	10.5	7	12.3	0	0.0
4+	10	17.5	0	0.0	0	0.0	0	0.0
Total	55	96.5	57	100.0	54	94.7	53	93.0

The density of CD3+, CD8+, CD45RO+ and FoxP3+ TILs was correlated with clinicopathological characteristics of our patients. Intratumoral FoxP3+ TILs were more often present in patients with single PM (χ^2^*P* = 0.011). All (10/10 (100 %) patients with a DFI of more than 60 months had high levels of CD45RO+ TILs at the invasive margin, compared to 24/36 (66.6 %) patients in the group DFI <36 months (DFI >60 vs. <36 months; Fisher’s exact test *P* = 0.044). FoxP3+ TILs at the invasive margin were associated with evidence for lymphatic vessel invasion (χ^2^ test *P* = 0.050). High CD3+ infiltration in the tumor center was more often found in PM from colon cancer compared to rectal cancer (χ^2^*P* = 0.040). Otherwise, no association of TILs density with age, sex, tumor localization, UICC stage of the primary tumor, previous liver metastasis or chemotherapy before metastasectomy was evident (Table 3).

**Table 3:** Association of CD3+, CD8+, CD45RO+ and FoxP3+ TILs in the tumor center and at the invasive margin with clinicopathological characteristics (significant values (*P* < 0.05) in bold)

	CD3+ TILs						CD8+ TILs					
	Tumor center			Invasive margin			Tumor center			Invasive margin		
	Low	High	*P*	Low	High	*P*	Low	High	*P*	Low	High	*P*
Age at surgery												
<64	12	15	0.346	9	18	0.210	21	7	0.473	24	4	0.423
≥64	16	12		14	14		24	5		27	2	
Sex												
Male	15	17	0.480	12	20	0.444	24	9	0.177	29	4	1.000
Female	13	10		11	12		21	3		22	2	
Localization of primary tumor												
Colon	12	19	**0.040**	13	18	0.984	23	9	0.138	28	4	0.686
Rectum	16	8		10	14		22	3		23	2	
UICC stage of primary tumor												
I	1	3	0.815	0	4	0.165	3	1	0.511	3	1	0.292
II	6	6		4	8		13	1		14	0	
III	15	12		15	12		20	7		23	4	
IV	4	5		3	6		7	2		8	1	
Unknown (n = 3)												
Previous liver metastasis												
No	17	22	0.090	18	21	0.309	30	10	0.315	35	5	0.657
Yes	11	5		5	11		15	2		16	1	
DFI												
<36 months	21	17	0.547	18	20	0.343	32	6	0.271	35	3	0.554
36–60 months	4	4		3	5		7	2		8	1	
60 months	3	6		2	7		6	4		8	2	
No. of pulmonary metastases												
Singular	17	18	0.646	13	22	0.352	29	8	1.000	32	5	0.410
Multiple	11	9		10	10		16	4		19	1	
Lymphatic vessel invasion												
No	20	14	0.135	15	19	0.660	29	6	0.506	31	4	1.000
Yes	8	13		8	13		16	6		20	2	
Chemotherapy before metastasectomy												
No	4	7	0.281	3	8	0.326	10	2	1.000	11	1	1.000
Yes	24	20		20	24		35	10		40	5	

	CD45RO+ TILs						FoxP3+ TILs					
	Tumor center			Invasive margin			Tumor center			Invasive margin		
	Low	High	*P*	Low	High	*P*	Low	High	*P*	Low	High	*P*
Age at surgery												
<64	10	17	0.580	6	21	0.362	15	12	0.494	19	8	0.697
≥64	12	15		9	18		12	14		17	9	
Sex												
Male	11	20	0.361	10	21	0.393	16	15	0.908	22	9	0.573
Female	11	12		5	18		11	11		14	8	
Localization of primary tumor												
Colon	13	8	0.836	11	20	0.142	16	15	0.908	22	9	0.573
Rectum	9	14		4	19		11	11		14	8	
UICC stage of primary tumor												
I	2	2	0.891	0	4	0.453	1	3	0.228	3	1	0.393
II	4	9		2	11		4	8		6	6	
III	11	15		8	18		17	9		20	6	
IV	3	5		3	5		4	4		6	2	
Unknown (n = 3)												
Previous liver metastasis												
No	14	24	0.369	9	29	0.333	16	21	0.088	23	14	0.172
Yes	8	8		6	10		11	5		13	3	
DFI												
<36 months	17	19	0.298	12	24	0.082	19	18	0.641	24	13	0.547
36–60 months	3	5		3	5		5	3		7	1	
60 months	2	8		0	10		3	5		5	3	
No. of pulmonary metastases												
Singular	15	21	0.845	10	26	1.000	14	22	**0.011**	22	14	0.122
Multiple	7	11		5	13		13	4		14	3	
Lymphatic vessel invasion												
No	14	19	0.752	11	22	0.253	17	15	0.695	25	7	**0.050**
Yes	8	13		4	17		10	11		11	10	
Chemotherapy before metastasectomy												
No	6	6	0.517	3	9	1.000	4	7	0.277	7	4	0.730
Yes	16	26		12	30		23	19		29	13	

*DFI* disease-free survival to first pulmonary metastasis

### Correlation of TILs and TLS in pulmonary metastases and corresponding primary CRC

A detailed description of CD3+, CD8+, CD45RO+ and FoxP3+ TILs density in the tumor center and at the invasive margin of the corresponding primary tumors is provided in Supplementary Table 4. We found no significant correlation between the TIL density in the primary tumor and corresponding lung metastases (Supplementary Table 5). In general, PM had higher densities of CD3+, CD8+, CD45RO+ TILs, whereas the FoxP3 TIL were comparable (Supplementary Fig. 1). Significantly less primary CRC were rated as TLS positive compared to the paired PM samples [2/28 (7.1 %) vs. 22/28 (78.6 %); McNemar test *P* < 0.001].

### Impact of TILs and TLS in PM on outcome parameters

**Fig. 2:**
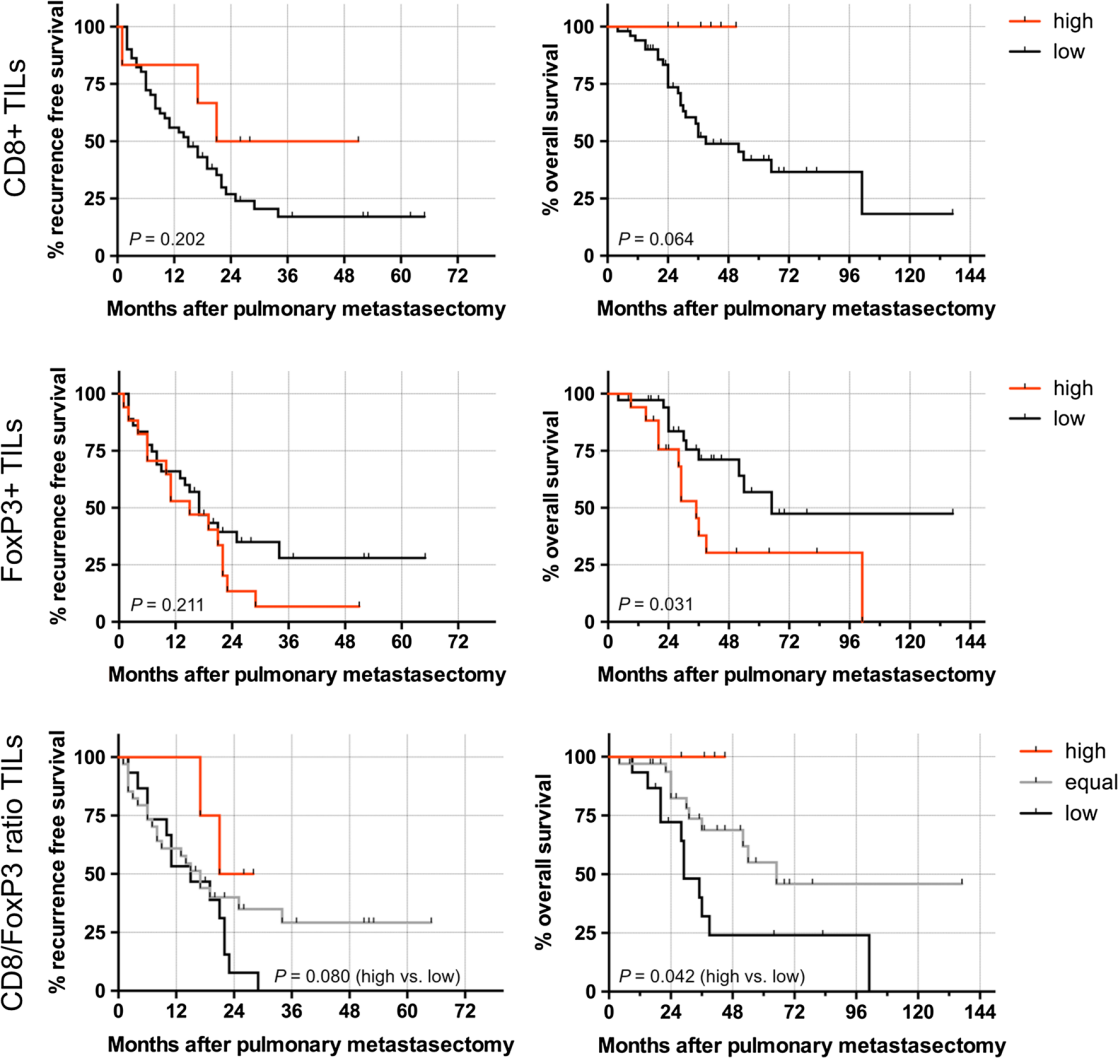
Kaplan–Meier estimates regarding recurrence-free survival and overall survival of pulmonary metastases dependent on the density of CD8+ and FoxP3+ TILs. Additionally the outcome for the CD8/FoxP3-ratio was calculated

The presence of FoxP3+ TILs at the invasive margin was significantly associated with a decreased OS (35 vs. 65 months; HR 2.40; 95 % CI 1.11–6.96; log-rank test *P* = 0.031) (Fig. 2). Moreover, dense CD8+ infiltrates at the invasive margin were associated with improved OS (median survival not reached vs. 39 months; HR 0.00; 95 % CI 0.09–1.04; log-rank test *P* = 0.064).

6/29 (20.7 %) patients had a high CD8/FoxP3-ratio, CD8/FoxP3 was equal in 10/29 (34.5 %) patients and a low ratio was found in 13/29 (44.8 %) patients. The CD8/FoxP3-ratio had significant impact on OS prognosis after pulmonary metastasectomy (log-rank test *P* = 0.021 and 0.042 for low vs. equal and low vs. high, respectively). Furthermore, patients with a high CD8/FoxP3-ratio at the invasive margin had a prolonged recurrence-free survival after pulmonary metastasectomy compared to patients with low CD8/FOXP3 ratio (26 vs. 15 months; HR 0.32; 95 % CI 0.12–1.03; log-rank test *P* = 0.080).

**Fig. 3:**
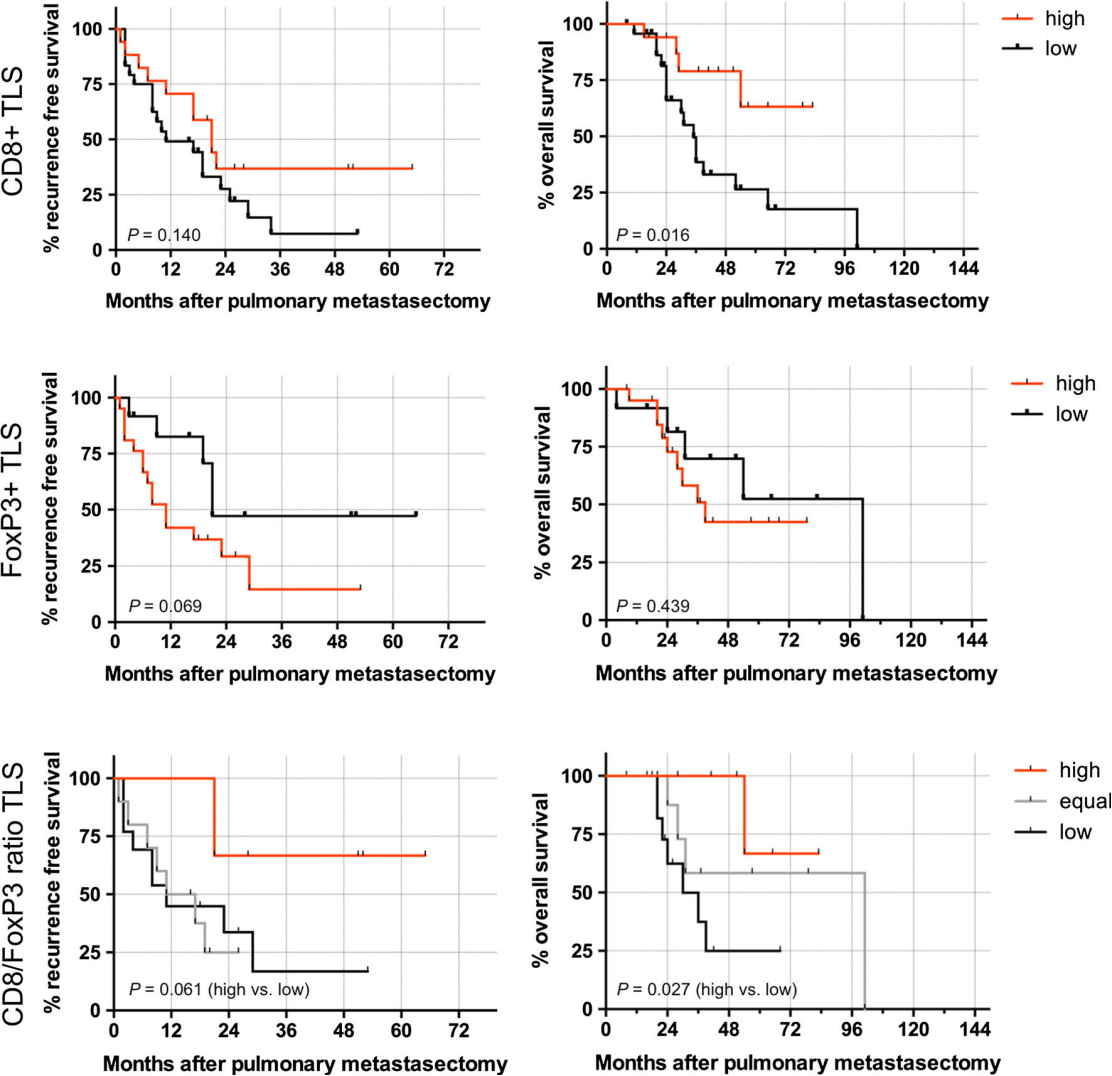
Kaplan–Meier estimates regarding recurrence-free survival and overall survival of pulmonary metastases dependent on the density of CD8+ and FoxP3+ cells in TLS. Additionally the outcome for the CD8/FoxP3-ratio was calculated

The presence of TLSs in PM alone was neither associated with recurrence-free survival (log-rank test *P* = 0.141) nor with OS (*P* = 0.813). However, when assessing the T cell subsets of TLS, we found a significant association of high levels of CD8+ cells in TLS and improved OS (median survival not reached vs. 35 months; HR 0.30; 95 % CI 0.14–0.79; log-rank test *P* = 0.016). Similarly to TILs, a strong prognostic effect was also found for the CD8/FOXP3-ratio in TLS (high vs. low HR 6.99; 95 % CI 1.28–20.48; log-rank test *P* = 0.027). Outcome analyses of CD3+, CD8+, CD45RO+ and FoxP3+ cells in TLS are summarized in Table 4 and Fig. 3. Adding CD8+ in TLS and FoxP3+ at the invasive margin into a multivariate Cox regression model, only CD8+ cells in TLS remained a significant prognosticator for OS after pulmonary metastasectomy (*P* = 0.020; HR 0.29; 95 % CI 0.07–0.79).

**Table 4:** Univariate and multivariate outcome analysis of recurrence-free survival and overall survival after pulmonary metastasectomy (significant values (*P* < 0.05) in bold)

	Recurrence-free survival			Overall survival				
	Univariate analysis (log-rank)			Univariate analysis (log-rank)			Multivariate Cox regression	
	Months	HR (95 % CI)	*P*-value	Months	HR (95 % CI)	*P*-value	HR (95 % CI)	*P*-value
Sex								
Male	21	0.65 (0.33–1.19)	0.516	54	1.30 (0.59–2.86)	0.516	–	–
Female	13.5			101			–	–
Age (years)								
<64	17	1.01 (0.54–1.88)	0.974	52	1.09 (0.50–2.40)	0.831	–	–
≥64	15			65			–	–
Location								
Colon	19	0.97 (0.52–1.80)	0.917	65	0.75 (0.34–1.62)	0.465	–	–
Rectum	11			36			–	–
UICC stage								
I + II	17	1.14 (0.59–2.24)	0.683	36	1.95 (0.88–4.85)	0.099	–	–
III + IV	15			NR			–	–
Unknown	3						–	–
Chemotherapy before metastasectomy								
Yes	15	1.19 (0.57–2.49)	0.648	52	0.81 (0.30–2.09)	0.644	–	–
No	22			65			–	–
Chemotherapy after metastasectomy								
Yes	15	1.34 (0.65–2.72)	0.443	39	2.08 (0.71–4.74)	0.214	–	–
No	21			NR			–	–
Previous liver metastasis								
Yes	9	1.99 (1.11–4.81)	**0.029**	36	1.39 (0.61–3.44)	0.414	–	–
No	21			54			–	–
DFI								
<36	17	1.20 (0.62–2.31)	0.600	52	1.15 (0.51–2.62)	0.741	–	–
≥36	17			65			–	–
No. of nodules								
Singular	17	0.93 (0.48–1.78)	0.811	54	0.85 (0.37–1.89)	0.681	–	–
Multiple	16			36			–	–
CD3+ TILs TC								
High	21	0.66 (0.35–1.23)	0.199	54	1.10 (0.48–2.53)	0.820	–	–
Low	15			52			–	–
CD3+ TILs IM								
High	16	1.46 (0.77–2.78)	0.254	54	1.68 (0.76–3.89)	0.213	–	–
Low	19			101			–	–
CD8+ TILs TC								
High	25	0.57 (0.29–1.24)	0.181	65	0.36 (0.17–1.27)	0.143	–	–
Low	15			39			–	–
CD8+ TILs IM								
High	43	0.48 (0.23–1.34)	0.202	NR	0.00 (0.09–1.04)	0.064	–	–
Low	15			39			–	–
CD45+ TILs TC								
High	17	0.88 (0.45–1.66)	0.676	65	0.78 (0.34–1.69)	0.515	–	–
Low	15			36			–	–
CD45+ TILs IM								
High	17	0.91 (0.44–1.84)	0.984	54	0.79 (0.32–1.78)	0.451	–	–
Low	17			36			–	–
FoxP3+ TILs TC								
High	17	0.99 (0.52–1.89)	0.982	54	0.98 (0.43–2.27)	0.969	–	–
Low	15			52			–	–
FoxP3+ TILs IM								
High	15	1.50 (0.79–3.16)	0.211	35	2.40 (1.11–6.96)	**0.031**	0.61 (0.23–1.61)	0.319
Low	17			65				
TLS								
Present	17	1.00 (0.48–2.10)	0.999	52	0.65 (0.26–1.71)	0.413	–	–
Not present	15			NR			–	–
CD3+ TLS								
High	21	1.85 (0.36–7.72)	0.525	65	0.34 (0.02–1.70)	0.136	–	–
Low	19			52			–	–
CD8+ TLS								
High	21	0.58 (0.27–1.18)	0.140	NR	0.30 (0.14–0.79)	**0.016**	4.39 (1.25–15.32)	**0.020**
Low	11			35				
CD45+ TLS								
High	17	1.05 (0.40–2.80)	0.918	65	1.03 (0.33–3.25)	0.949	–	–
Low	17			30			–	–
FoxP3+ TLS								
High	11	2.42 (0.96–5.59)	0.069	39	1.50 (0.54–4.45)	0.439	–	–
Low	21			101			–	–
CD8/FoxP3-ratio IM								
Low	15	1		29	1		–	–
Equal	17	0.65 (0.30–1.26)	0.197	65	0.39 (0.13–0.83)	**0.021**	–	–
High	26	0.32 (0.12–1.03)	0.080	NR	n/a	**0.042**	–	–
CD8/FoxP3-ratio TLS								
Low	11	1		30	1		–	–
Equal	14	0.89 (0.32–2.37)	0.804	101	2.13 (0.71–7.60)	0.191	–	–
High	NR	3.65 (1.02–10.96)	0.061	NR	6.99 (1.28–20.48)	**0.027**	–	–

*DFI* disease-free survival to first pulmonary metastasis, *IM* invasive margin, *NR* 50 % recurrence/survival not reached, *TC* tumor center, *TILs* tumor-infiltrating lymphocytes, *TLS* tertiary lymphoid structure

## Discussion

The aim of this study was to evaluate the role of TILs and TLSs in PM assessing a cohort of patients with CRC lung metastases. CD3+ TILs were found in every resected pulmonary metastatic specimen, highlighting the pivotal role of the adaptive immune system in local tumor microenvironment. We could show that tumor infiltrating CD8+ and FoxP3 positive cells were associated with disease free survival after pulmonary metastasectomy and OS.

CD8+ T cells represent a subpopulation of T cells, also known as cytotoxic T cells. They play an important role in the defense against viruses but also cancer cells. Upon activation they release cytotoxins (e.g., perforin, granzymes, granulysin) into infected or tumorous somatic cells, which eventually leads to the induction of apoptosis. Tumor infiltrating CD8+ cells can induce a potent tumorlytic response, which has been shown for various malignancies [25].

FoxP3+ cells are known as regulatory T-cells (Tregs). The have the ability to suppress effector T-cell function both in a paracrine and cell–cell-contact dependent manner [26]. Tregs are important for the maintenance of immunological tolerance, however, can also dampen antitumor response of the immune system. An expansion of the Treg pool experimentally leads to enhanced vulnerability of carcinogens and worse outcome [27, 28].

The role of TILs has been extensively studied in primary CRC. The inflammatory infiltrate was shown to correlate with the T-stage of primary CRC and even allowed a more precise prognosis on patients’ outcome compared to the UICC–TNM staging alone [10]. In the subgroup of rectal cancer patients, the prognostic value of the immune infiltrate (CD3 and CD8) was confirmed and additionally found to be a predictive marker for the response to preoperative chemo-radiotherapy [9]. Based on this data, an international consortium was founded to standardize and implement an adapted staging system taking the immune infiltrate (“Immunoscore”) into account [29].

In contrast to the evidence of the role of TILs in primary CRC, sparse data exists on TILs in lung metastases. To the best of our knowledge, by now only two studies have examined TILs in CRC lung metastases. Remark et al. showed in a retrospective cohort with CRC PM that a high density of CD8+ TILs conferred an improved OS (*P* = 0.039 in univariate analysis). Interestingly, CD8+ TILs at the tumor center and at the invasive margin had a similar prognostic impact [30]. In another study conducted in a Korean patient cohort with UICC stage IV colon cancer, including 21 patients with PM and 58 with liver metastases, a high CD8+ TILs density in the primary tumor, but not in the metastatic tissue (liver and lung together) had a beneficial impact on OS (log-rank test *P* = 0.017 and 0.232, respectively). The strongest positive prognostic impact was found for high CD45RO+ TILs in the primary and metastatic tissue (log-rank test *P* = 0.009 and 0.027, respectively). The prognostic impact of CD45RO+ TILs in the primary tumor remained significant in a multivariate model (RR 0.108; 95 % CI 0.021–0.546; *P* = 0.007). Interestingly, a high density of FoxP3+ TILs at the metastatic site was also associated with a favorable prognosis (log rank test *P* = 0.050) in this study [31]. This is in contrast to most of the published evidence on immunosurveillance in CRC and this finding could not be confirmed in our patient cohort [32, 33]. This discrepancy might be explained by the mixture of liver (73 %) and lung metastases (27 %), which were not separated in the outcome analysis [31]. Another explanation might be the spatial distribution of FoxP3 cells. Salama et al. reported a decreased survival of stage II CRC patients with low content of FoxP3+ T-cells within the tumor (HR 0.65; 95 % CI 0.48–0.89; *P* = 0.007), whereas the opposite was found when assessing adjacent colonic tissue (HR 1.42; 95 % CI 1.05–1.92; *P* = 0.023) [11]. The authors speculated that FoxP3+ T-regulatory cells might lose their ability to suppress antitumor immunity when they are found within the tumor tissue. Our results suggest differing impact of FoxP3+ TILs depending on their localization. Dense infiltration of FoxP3+ TILs in the infiltration zone was associated with impaired survival, whereas infiltration of FoxP3+ TILs in the tumor center did not alter survival. Moreover, high levels of FoxP3+ TILs at the invasive margin were significantly more common in patients with evidence for lymphatic invasion, which is believed to be a key-determinant of the outcome after pulmonary metastasectomy [19, 31]. Similar to two recent publications, which described the ratio between CD8+ and FoxP3+ TILs as prognostic factor superior to CD8+ and FoxP3+ TILs alone, we found the CD8/FoxP3- ratio to have a strong impact on OS in our patient cohort [34, 35]. In general, our observations in PMs imply differential roles of TILs dependent on their localization within the metastatic tissue. This has to be taken in consideration when assessing TILs in histological specimen and when defining cut-offs for statistical evaluation.

The present work is, to the best of our knowledge, the first analysis of presence and composition of TLS in CRC lung metastases. We could demonstrate that high levels of CD8+ cells as well as a high CD8/FoxP3-ratio in TLS correlated positively with patients’ OS after pulmonary metastasectomy (*P* = 0.016 and 0.027, respectively). Similar to our findings on TILs at the invasive margin, the balance of CD8+ and FoxP3+ in TLS seems to be a crucial factor for the survival of CRC patients with dissemination to the lung. Di Caro et al. found that TLS were present in 78.6 % of stages II and III primary CRC. The presence of TLS was a positive prognosticator only in stage II CRC (log rank *P* = 0.02), whereas the prognostic impact was lost in an advanced tumor stage (UICC stage III) [16]. Also in our cohort of stage IV CRC patients, the presence of TLS alone had no impact on recurrence-free and OS. Salama et al. evaluated the distribution of FoxP3+ cells in TLS in stage II colon cancer and described the high density of FoxP3+ cells in TLS as negative prognostic factor of OS (HR 4.22; 95 % CI 1.49–11.91; *P* = 0.007 in multivariate analysis) [36]. In our cohort, patients with high number of FoxP3+ cells in TLS evidenced a decreased recurrence-free and OS, without reaching the level of significance. Only 7.1 % of the primary CRC samples were rated as TLS positive in our cohort. This might indicate an insufficient immune response at the primary tumor site in patients subsequently developing metastatic disease. However, caution is warranted when interpreting this finding due to the small sample size.

Interestingly, the density of the immune infiltrate in primary tumors did not correlate with the infiltrate in PM. Considering the heterogeneity of tumors, the metastatic spreading of subpopulations of tumor cells of the primary cancer to distant organs might explain the different immuno-phenotype observed in our cohort [37]. In general, lung metastases appear to consist of more immunogenic tumor cells compared to the primary tumor site, since the density of CD3+, CD8+ and CD45+ TIL increased during progression of disease. Another possible explanation for this observation might be the unique immunological state of the lung. It is constantly exposed to environmental pathogens and therefor rich in residing immune cells.

Our findings suggest that patients with CRC lung metastases is a heterogeneous group regarding the tumor biology. This might have implications on the management of these patients. Histological evidence for an aggressive tumor biology might have consequences regarding the offered adjuvant chemotherapy, tumor surveillance strategies and a possible future re-metastasectomy.

There are several limitations to this study. First, no uniform protocol for pseudo-neoadjuvant and pseudo-adjuvant chemotherapeutic regimens was applied. According to the preferences of the referring oncologist, different schemes of chemotherapy were administered to the patients. As chemotherapeutic agents might contribute to the immune-editing of tumors, this is a possible confounder of this study. However we could not find a significant association between administration of chemotherapy before metastasectomy and the immune infiltrate in PMs. Moreover, the history of chemotherapy before metastasectomy did not affect the outcome after lung metastasectomy. Second, patients eligible for surgery are a subgroup and do not represent the whole population of patients with lung metastases from CRC. Thus, our findings might not be extrapolated to all patients with CRC PMs due to this selection bias. Last, although this is currently the biggest cohort of patients with CRC PM in which the CD3+, CD8+, CD45RO+ and FoxP3+ TILs and TLS was assessed, the study might still be underpowered. A multi-institutional study with a considerable sample size will be necessary to further clarify the prognostic value of TILs and TLS in CRC lung metastases.

In summary, this is the first structured analysis of CD3+, CD8+, CD45RO+ and FoxP3+ TILs and TLS in the setting of curative pulmonary metastasectomy, including more than 50 patients with CRC lung metastases. Our results suggest that especially the balance of effector CD8+ and regulatory FoxP3+ TILs play a crucial role during immune-editing of metastatic CRC and thus, predicting outcome of patients after pulmonary metastasectomy with curative intent.

## Electronic supplementary material

Below is the link to the electronic supplementary material.

Supplementary material 1 (TIFF 14823 kb)

Supplementary material 2 (XLSX 41 kb)

Supplementary material 3 (DOCX 75 kb)

Supplementary Table 1 Cut-offs used for dichotomization

Supplementary Table 2 Semi-quantitative description of CD3+, CD8+, CD45RO+ and FoxP3+ TLS in CRC pulmonary metastases

Supplementary Table 3 Association of CD3+, CD8+, CD45RO+ and FoxP3+ TLS with clinicopathological characteristics. The number of samples in which TLS could be found is provided in the first row 26

Supplementary Table 4 Semi-quantitative description of tumor-infiltrating lymphocytes in corresponding primary colorectal cancer (n = 31)

Supplementary Table 5 Spearman’s rho and P-values comparing the semiquantitative CD3+, CD8+, CD45RO+ and FoxP3+ TIL density in primary CRC and corresponding pulmonary metastases

Supplementary Fig. 1 Comparison of CD3+, CD8+, CD45RO+ and FoxP3+ TILs in CRC pulmonary metastases (m) and primary tumors (p) in percent
