# Supplementary figures and images for: Tumor-infiltrating lymphocyte subsets and tertiary lymphoid structures in pulmonary metastases from colorectal cancer
Source: Clin Exp Metastasis. 2016 Jul 23;33(7):727–39. doi: 10.1007/s10585-016-9813-y (PMC5035322; doi:10.1007/s10585-016-9813-y)

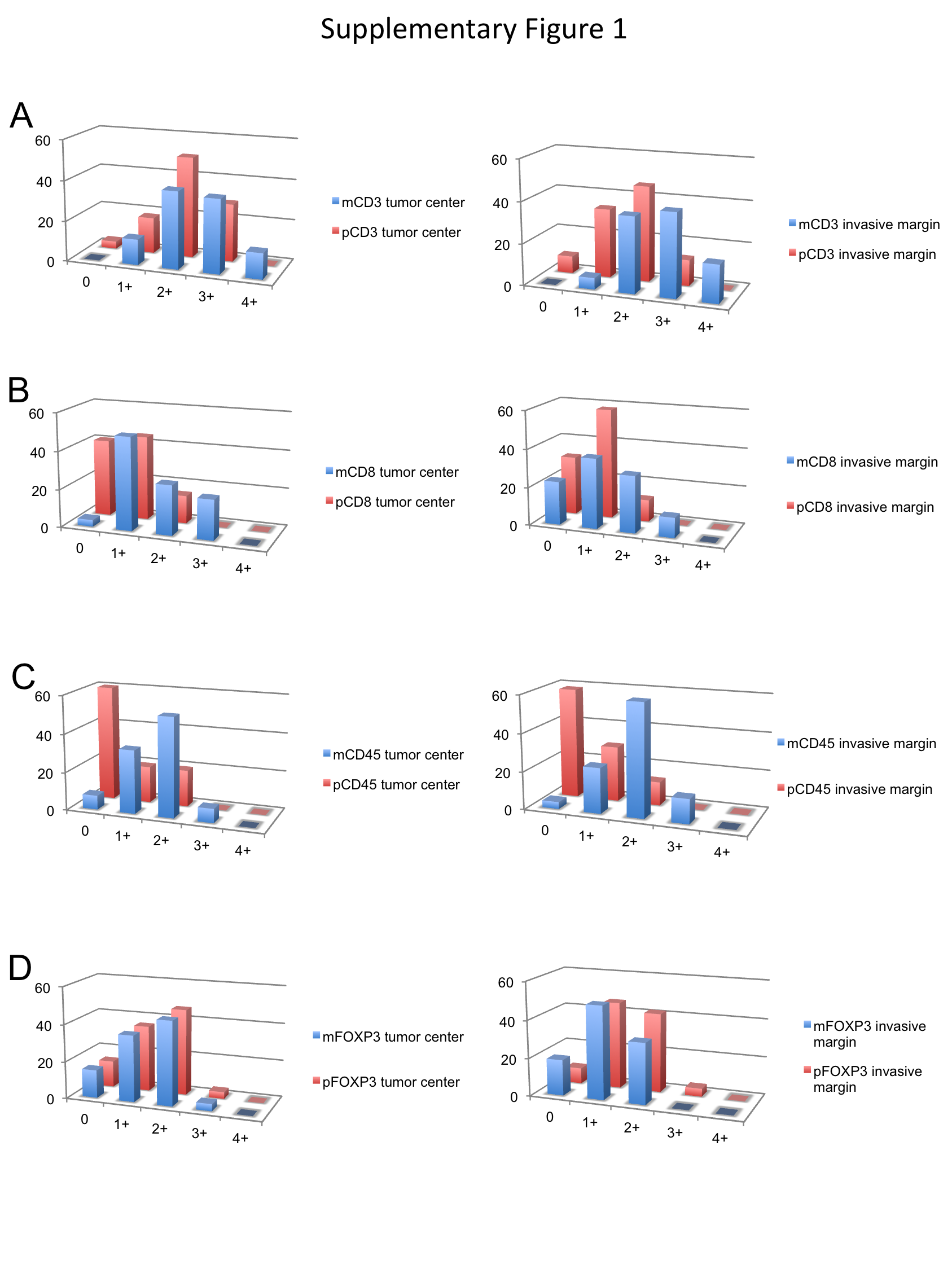

Supplement: Supplementary file 1 — Supplementary material 1 (TIFF 14823 kb) [file 10585_2016_9813_MOESM1_ESM.tiff]
