# Supplementary material for: Tumor-infiltrating lymphocyte subsets and tertiary lymphoid structures in pulmonary metastases from colorectal cancer
Source: Clin Exp Metastasis. 2016 Jul 23;33(7):727–39. doi: 10.1007/s10585-016-9813-y (PMC5035322; doi:10.1007/s10585-016-9813-y)
Supplement: Supplementary file 3 — Supplementary material 3 (DOCX 75 kb) [file 10585_2016_9813_MOESM3_ESM.docx]

**Supplementary Table 1**

|  |  | Primary CRC | Pulmonary metastases |
| --- | --- | --- | --- |
|  | CD3 tumor center | 0 1+ 2+ II 3+ 4+ | 0 1+ 2+ II 3+ 4+ |
|  | CD3 tumor border | 0 1+ 2+ II 3+ 4+ | 0 1+ 2+ II 3+ 4+ |
|  | CD8 tumor center | 0 1+ II 2+ 3+ 4+ | 0 1+ 2+ II 3+ 4+ |
|  | CD8 tumor border | 0 1+ 2+ II 3+ 4+ | 0 1+ 2+ II 3+ 4+ |
| TILs | CD45RO tumor center | 0 1+ II 2+ 3+ 4+ | 0 1+ II 2+ 3+ 4+ |
|  | CD45RO tumor border | 0 1+ II 2+ 3+ 4+ | 0 1+ II 2+ 3+ 4+ |
|  | FoxP3 tumor center | 0 1+ II 2+ 3+ 4+ | 0 1+ II 2+ 3+ 4+ |
|  | FoxP3 tumor border | 0 1+ II 2+ 3+ 4+ | 0 1+ II 2+ 3+ 4+ |
|  | CD3 | 0 1+ 2+ II 3+ 4+ | 0 1+ 2+ II 3+ 4+ |
|  | CD8 | 0 1+ II 2+ 3+ 4+ | 0 1+ II 2+ 3+ 4+ |
| TLS | CD45RO | 0 1+ II 2+ 3+ 4+ | 0 1+ II 2+ 3+ 4+ |
|  | FoxP3 | 0 1+ II 2+ 3+ 4+ | 0 1+ II 2+ 3+ 4+ |

**Supplementary Table 2**

|  | **CD3+ TLS** | | **CD8+ TLS** | | **CD45RO+ TLS** | | **FoxP3+ TLS** | |
| --- | --- | --- | --- | --- | --- | --- | --- | --- |
|  | **n** | **%** | **n** | **%** | **n** | **%** | **n** | **%** |
| **w/o TLS** | 18 | 32.7 | 16 | 28.1 | 16 | 32.0 | 16 | 32.7 |
| **w/ TLS** | **37** | **67.3** | **41** | **71.9** | **34** | **68.0%** | **33** | **67.3** |
| None | 0 | 0.0 | 3 | 5.3 | 1 | 2.0 | 2 | 4.1 |
| Sparse | 0 | 0.0 | 21 | 36.8 | 7 | 14.0 | 10 | 20.4 |
| Moderate | 4 | 7.3 | 11 | 19.3 | 15 | 30.0 | 20 | 40.8 |
| Dense | 21 | 38.2 | 6 | 10.5 | 11 | 22.0 | 1 | 2.0 |
| Very dense | 12 | 21.8 | 0 | 0.0 | 0 | 0.0 | 0 | 0.0 |

**Supplementary Table 3**

|  | CD3+ TLS  n=37 | | | CD8+ TLS  n=41 | | | CD45RO+ TLS  n=34 | | | FoxP3+ TLS  n=33 | | |
| --- | --- | --- | --- | --- | --- | --- | --- | --- | --- | --- | --- | --- |
|  | low | high | *P=* | low | high | *P=* | low | high | *P=* | low | high | *P=* |
| Age at surgery |  |  |  |  |  |  |  |  |  |  |  |  |
| <64 | 2 | 15 | 1.000 | 10 | 11 | 0.146 | 2 | 13 | 0.257 | 6 | 18 | 0.506 |
| ≥64 | 2 | 18 |  | 14 | 6 |  | 6 | 13 |  | 6 | 13 |  |
| Sex |  |  |  |  |  |  |  |  |  |  |  |  |
| Male | 2 | 15 | 1.000 | 14 | 9 | 0.732 | 5 | 15 | 1.000 | 7 | 12 | 0.947 |
| Female | 2 | 18 |  | 10 | 8 |  | 3 | 11 |  | 5 | 9 |  |
| Localization of primary tumor |  |  |  |  |  |  |  |  |  |  |  |  |
| Colon | 4 | 20 | 0.276 | 15 | 9 | 0.540 | 5 | 15 | 1.000 | 6 | 11 | 0.895 |
| Rectum | 0 | 13 |  | 9 | 8 |  | 3 | 11 |  | 6 | 10 |  |
| UICC stage of primary tumor |  |  |  |  |  |  |  |  |  |  |  |  |
| I | 0 | 2 | 0.305 | 1 | 2 | 0.124 | 0 | 2 | 0.903 | 2 | 1 | 0.035 |
| II | 0 | 8 |  | 9 | 2 |  | 3 | 6 |  | 0 | 8 |  |
| III | 2 | 17 |  | 9 | 11 |  | 3 | 13 |  | 6 | 9 |  |
| IV | 2 | 4 |  | 4 | 1 |  | 1 | 4 |  | 3 | 2 |  |
| unknown (N=3) |  |  |  |  |  |  |  |  |  |  |  |  |
| Previous liver metastasis |  |  |  |  |  |  |  |  |  |  |  |  |
| No | 3 | 25 | 1.000 | 16 | 14 | 0.309 | 7 | 20 | 1.000 | 8 | 19 | 0.159 |
| Yes | 1 | 8 |  | 8 | 3 |  | 1 | 6 |  | 4 | 2 |  |
| DFI |  |  |  |  |  |  |  |  |  |  |  |  |
| <36 months | 2 | 23 | 0.391 | 15 | 11 | 0.188 | 6 | 16 | 0.848 | 7 | 15 | 0.643 |
| 36-60 months | 1 | 4 |  | 6 | 1 |  | 1 | 4 |  | 2 | 2 |  |
| 60 months | 1 | 6 |  | 3 | 5 |  | 1 | 6 |  | 3 | 4 |  |
| No. of pulmonary metastases |  |  |  |  |  |  |  |  |  |  |  |  |
| singular | 3 | 22 | 1.000 | 14 | 12 | 0.422 | 5 | 19 | 0.666 | 8 | 17 | 0.420 |
| multiple | 1 | 11 |  | 10 | 5 |  | 3 | 7 |  | 4 | 4 |  |
| Lymphatic vessel invasion |  |  |  |  |  |  |  |  |  |  |  |  |
| no | 1 | 22 | 0.142 | 12 | 13 | 0.087 | 6 | 13 | 0.257 | 7 | 9 | 0.392 |
| yes | 3 | 11 |  | 12 | 4 |  | 2 | 13 |  | 5 | 12 |  |
| Chemotherapy before metastasectomy |  |  |  |  |  |  |  |  |  |  |  |  |
| No | 2 | 7 | 0.244 | 6 | 3 | 0.711 | 2 | 7 | 1.000 | 2 | 6 | 0.678 |
| Yes | 2 | 26 |  | 18 | 14 |  | 6 | 19 |  | 10 | 15 |  |

**Supplementary Table 4**

|  | **CD3+ TIL** | | **CD8+ TIL** | | **CD45RO+ TIL** | | **FoxP3+ TIL** | |
| --- | --- | --- | --- | --- | --- | --- | --- | --- |
| **TIL density** | **n** | **%** | **n** | **%** | **n** | **%** | **n** | **%** |
| **Tumor center** |  |  |  |  |  |  |  |  |
| 0 | 1 | 3.3 | 11 | 35.4 | 16 | 51.6 | 4 | 12.9 |
| 1+ | 5 | 16.1 | 12 | 38.7 | 5 | 16.1 | 10 | 32.2 |
| 2+ | 14 | 45.2 | 4 | 12.9 | 5 | 16.1 | 13 | 41.9 |
| 3+ | 8 | 25.8 | 0 | 0.0 | 0 | 0.0 | 1 | 3.3 |
| 4+ | 0 | 0.0 | 0 | 0.0 | 0 | 0.0 | 0 | 0.0 |
| **Total** | **28** | **90.3** | **27** | **87.1** | **26** | **83.9** | **28** | **90.3** |
| **Invasive margin** |  |  |  |  |  |  |  |  |
| 0 | 2 | 6.4 | 8 | 25.8 | 14 | 45.1 | 3 | 6.4 |
| 1+ | 8 | 25.7 | 15 | 48.4 | 7 | 22.6 | 11 | 35.4 |
| 2+ | 11 | 35.4 | 3 | 9.6 | 3 | 9.7 | 10 | 32.3 |
| 3+ | 3 | 9.7 | 0 | 0.0 | 0 | 0.0 | 1 | 3.3 |
| 4+ | 0 | 0. | 0 | 0.0 | 0 | 0.0 | 0 | 0.0 |
| **Total** | **24** | **77.4** | **26** | **83.9** | **24** | **77.4** | **24** | **77.4** |

**Supplementary Table 5**

|  | | Primary Tumor | | | | | | | |
| --- | --- | --- | --- | --- | --- | --- | --- | --- | --- |
|  |  | CD3 tumor center | CD3 tumor border | CD8 tumor center | CD8 tumor border | CD45RO tumor center | CD45RO tumor border | FoxP3 tumor center | FoxP3 tumor border |
| Lung metastases | CD3 tumor center | 0.129  P=0.514 |  |  |  |  |  |  |  |
|  | CD3 tumor border |  | 0.261  P=0.218 |  |  |  |  |  |  |
|  | CD8 tumor center |  |  | 0.037  P=0.855 |  |  |  |  |  |
|  | CD8 tumor border |  |  |  | 0.171  P=0.404 |  |  |  |  |
|  | CD45RO tumor center |  |  |  |  | -0.334  P=0.103 |  |  |  |
|  | CD45RO tumor border |  |  |  |  |  | 0.052  P=0.812 |  |  |
|  | FoxP3 tumor center |  |  |  |  |  |  | 0.006  P=0.975 |  |
|  | FoxP3 tumor border |  |  |  |  |  |  |  | 0.106  P=0.632 |
